# Mechanisms linking the human gut microbiome to prophylactic and treatment strategies for COVID-19

**DOI:** 10.1017/S0007114520003980

**Published:** 2020-10-09

**Authors:** Gemma E. Walton, Glenn R. Gibson, Kirsty A. Hunter

**Affiliations:** 1 Food Microbial Sciences Unit, Department of Food and Nutritional Sciences, University of Reading, Reading RG6 6AP, UK; 2 Exercise and Health Research Group, Department of Sport Science, Sport, Health and Performance Enhancement (SHAPE) Research Centre, Nottingham Trent University, Nottingham NG11 8NS, UK

**Keywords:** Coronavirus, COVID-19, Gut microbiome, Probiotics, Prebiotics, ACE2, angiotensin-converting enzyme 2, LRTI, lower respiratory tract infection, URTI, upper respiratory tract infection

## Abstract

The recent COVID-19 pandemic has altered the face of biology, social interaction and public health worldwide. It has had a destructive effect upon millions of people and is approaching a devastating one million fatalities. Emerging evidence has suggested a link between the infection and gut microbiome status. This is one of the several factors that may contribute towards severity of infection. Given the fact that the gut is heavily linked to immunity, inflammatory status and the ability to challenge pathogens, it is worthwhile to consider dietary intervention of the gut microbiota as means of potentially challenging the viral outcome. In this context, probiotics and prebiotics have been used to mitigate similar respiratory infections. Here, we summarise links between the gut microbiome and COVID-19 infection, as well as propose mechanisms whereby probiotic and prebiotic interventions may act.

At the end of 2019, an emerging viral illness was identified in Hubei Province, China. On 7 January 2020, a novel coronavirus, SARS-CoV-2, was isolated from a patient's respiratory sample. WHO announced ‘COVID-19’ as the name of this new disease on 11 February 2020. COVID-19 incidence escalated across the world and, by 12 March 2020, the WHO declared a COVID-19 pandemic. Since then, an epidemiological account has unfolded of a virus that has threatened global health and attacked world economy.

To date (15 September 2020), over 29·5 million people are known to have contracted COVID-19 worldwide and, devastatingly, over 933 000 have lost their lives^(1)^. Transmission of the virus has been rapid and, whilst some individuals have contracted a mild-to-moderate upper respiratory tract illness, others have faced much more serious manifestations including multiple organ failure and death. By looking into the profile of those hardest hit by the virus, lessons may be learnt and potential strategies for reducing the burden proposed. In this review, ways in which the gut microbiota may influence risk of contracting SARS-CoV-2 are considered along with how this could impact upon disease development in terms of severity and duration. Possible mechanisms within this interaction are considered along with evidence to support the use of gut microbiota as a potential prophylactic strategy.

The gut microbiome is the totality of the mixed community of micro-organisms, including genetic components, microbial biodiversity and their resulting functionality. Recent studies using metagenomic approaches have highlighted the complex inter-relationship between resident intestinal organisms and mammalian metabolism and have shown that the gut microbiota plays an important role not only in the way we derive energy from our diet but also in the manner in which we store this energy. Such studies have also identified roles for the gut microbiota in the aetiology and/or maintenance of gut disorders. Large sequencing projects, such as the MetaHit^(2)^ and the Human Microbiome project^(3)^, have helped to unravel new diversity as well as understand the composition of the microbiota in different clinical states. With accumulating evidence surrounding COVID-19 and the microbiota, this review brings together evidence from recent studies, contrasted with information about the gut microbiota and how it might be able to help in the fight against COVID-19 infection.

There are several mechanisms whereby the gut microbiota may influence viral transmission and disease progression. In relation to initial infection, Zuo *et al.* ^(4)^ compared the faecal microbiota of fifteen COVID-19 patients to healthy controls. When focusing on the microbiota of the seven antibiotic-naïve COVID-19 patients on admission to hospital, microbial sequencing revealed elevated levels of *Coprobacillus* spp. *Clostridium ramosum* and *Clostridium hathewayi* were associated with severity of COVID-19 symptoms along with reduced levels of *Alistipes* spp. and the anti-inflammatory associated with *Faecalibacterium prausnitzii*. It has been observed that COVID-19 gains entry to cells through angiotensin-converting enzyme 2 (ACE2) receptors^(5)^. ACE2 is a transmembrane protein that counteracts ACE, and its receptors are found within epithelium cells of the gut^(6)^. *Coprobacillus* spp. have been observed to up-regulate ACE2 in the murine gut^(7)^; thus, changes in the gut microbiota may alter ability of the virus to gain cellular entry into the gut. Indeed, positive virus staining has been observed in intestinal ACE-2 viral host cells^(8)^. It is also noteworthy that COVID-19 RNA has been found in faeces^(9)^. This, therefore, indicates viral replication as being likely within the intestine. However, whilst viral RNA has been found within faeces, to date, the authors are unaware of any studies where infectious viral particles have been recovered^(10)^. This could, in part, be a consequence of enteric secretions emulsifying the virus and rendering it inactive^(11)^. This means that whilst the virus may replicate within the large intestine, there is unlikely to be faecal–oral transmission. Internally, this could make integrity of the gut wall important for avoiding viral transfer.

Gu *et al.* ^(12)^ reported the presence of more potential pathogens in the gut microbiota of thirty hospitalised COVID-19 patients compared with healthy controls. Furthermore, Zuo *et al.* ^(13)^ used RNA shotgun metagenomics on faecal viral extractions to determine those with high and low SARS-CoV-2 infectivity in fifteen hospitalised COVID-19 patients. It was observed that patients with high infectivity had more potential pathogenic bacteria in their faeces than those with lower levels. It is important to consider the relevance this may have to the illness that ensues. The microbial community residing on mucosal surfaces of the gastrointestinal tract has both direct and indirect effects upon the host immune system (it is estimated that 70 % of the immune system is located within the gastrointestinal tract)^(14)^ and thus is a key player in defence against microbial infection. Research from Yaron *et al.* ^(15)^ using antibiotics and peptides to modulate the microbiota demonstrated importance of the microbiota in murine immune response to gammaherpesvirus-68. Indeed, impact of the gut microbiota on the body’s immune function is well evidenced, both in healthy and pathological conditions^(16)^. It is worth noting that COVID-19 progression appears to be associated with a cytokine storm that underpins hyper-inflammation, whereby elevated levels of pro-inflammatory cytokines, including TNF, IL-6 and IL-1β, are observed^(17)^. Approaches to combat this could aid in the reduction of symptom severity.

## Modulation of the gut microbiome through diet

Generally, the various components of the large intestinal microbiota may be considered as exerting pathogenic or potential health promoting effects. Bacteria in the colon respond largely to the available fermentable substrate, which is mainly provided by diet^(18)^. Through the process of fermentation, gut bacteria metabolise various substrates (principally dietary components) to form end products such as SCFA and gases. This anaerobic metabolism is thought to contribute positively towards host daily energy requirements. Fermentation by gut bacteria consists of a series of energy-yielding reactions that do not use oxygen in the respiratory chains. The electron acceptors may be organic (e.g. some products of the fermentation) or inorganic (e.g. sulphate, nitrate). As carbohydrates form the principal precursors for fermentation, ATP is usually formed through substrate level phosphorylation by saccharolytic micro-organisms. In terms of end products, a variety of different metabolites arise. Predominant of these are the SCFA acetate, propionate and butyrate.

In the gut, resilience is connected to the functional core microbiota^(19)^. Usually, the human host lives in harmony with its complex gut microbiota. However, under certain circumstances like antimicrobial intake, stress, poor diet and living conditions, the relationship may be compromised. The gut microbiota is also susceptible to contamination from transient pathogens, which further upsets the normal community structure. These factors can have consequences that may result in the onset of gut disorder, which can manifest through both acute and chronic means^(20–22)^. Dietary modulation of the gut microbiota is a functional food concept used to mitigate this.

### Probiotics

The first records of ingestion of live bacteria by humans are over 2000 years old^(23)^. However, at the beginning of the last century, probiotics were first put onto a scientific basis by the work of Metchnikoff at the Pasteur Institute in Paris. Metchnikoff^(24)^ hypothesised that the normal gut microbiota could exert adverse effects on the host and that consumption of ‘soured milks’ could help. This was the birth of the probiotic concept as we now know it. A formal probiotic definition is shown in Table 1. This implies that health outcomes should be defined and proven, which is not an easy task. Most research has been directed towards the use of intestinal isolates of bacteria as probiotics. Over the years, many types of micro-organisms have been used. They consist of not only lactic acid bacteria (lactobacilli, streptococci, enterococci, lactococci and bifidobacteria) but also *Bacillus* spp., *Escherichia coli* and yeasts such as *Saccharomyces* spp.^(25)^.

**Table 1.** Definitions and main points about probiotics, prebiotics and synbiotics

Approach	Definition	Main points	References
Probiotic	Live micro-organisms that, when administered in adequate amounts, confer a health benefit on the host	Probiotics must have been shown in well-controlled studies to confer benefits to health	25
		Commensals from human samples, with adequate evidence, can be probiotics	
		Live cultures associated with fermented foods but have no evidence of a health benefit are not probiotics	
		Undefined faecal transplants are not probiotics	
Prebiotic	A substrate that is selectively utilised by host micro-organisms conferring a health benefit	Most prebiotics are given orally and target the gut microbiota although other sites such as the vaginal tract, oral cavity and skin are under investigation	35
		Health benefits include cardiometabolism, mental health and bone	
		Currently established prebiotics are carbohydrate-based, but other substances such as polyphenols and PUFA may evolve	
		Beneficial effect(s) of a prebiotic on health must be confirmed in the host for its intended use	
Synbiotic	A mixture, comprising live micro-organisms and substrate(s) selectively utilised by host micro-organisms, which confers a health benefit on the host	Host micro-organisms include both autochthonous and allochthonous micro-organisms (such as probiotics)	36
		A complementary synbiotic is a mixture of a probiotic plus prebiotic	
		A synergistic synbiotic is composed of a live microbe(s) and a selectively utilised substrate(s), but neither needs to meet the minimum criteria stipulated previously for probiotics and prebiotics	

The actions of probiotics are usually strain specific and, generally speaking, main positive effects are associated with protection against gastroenteritis, improved lactose tolerance, stimulation of the immune system through non-pathogenic means, influencing atopic conditions and reductions in blood lipids^(26–28)^. Probiotic use in animals may take the form of powders, tablets, sprays and pastes. In humans, the most commonly used vector involves fermented milk products and ‘over the counter’ freeze-dried preparations of lactic acid bacteria in capsules. Recently, the market has expanded to include other foods such as flavoured drinks and pharmaceutical preparations such as tablets.

### Prebiotics

Prebiotics allow the selective growth of certain indigenous micro-organisms in a given ecosystem (Table 1). In the gut, an effective prebiotic ingredient should:

1. Neither be hydrolysed nor absorbed in the upper part of the gastrointestinal tract
2. Have a selective fermentation such that the composition of the large intestinal microbiota is altered towards a healthier composition.

The prebiotic concept has been derived to specifically increase the many positive micro-organisms, such as bifidobacteria and lactobacilli, already present in the human colon^(29)^. However, as knowledge of gut microbiota diversity has expanded, there may be other target genera such as *Roseburia, Eubacterium, Faecalibacterium, Akkermansia, Christensenella* and *Propionibacteria*, as has been discussed by Satokari^(30)^ and Chang *et al.* ^(31)^. It is the case, however, that more physiological understanding of these groups is required and their definitive health bonuses need to be more thoroughly understood before they can be advocated as prebiotic responders^(32)^.

Fructo-oligosaccharides and galacto-oligosaccharides are the most widely researched prebiotics^(33)^. Some prebiotics (inulin type fructans) occur naturally in several foods such as leek, asparagus, chicory, Jerusalem artichoke, garlic, artichoke, onion and banana. However, overall intake from these sources within a normal, in particular Western-type diet, is small. An effective route to achieve a health-promoting intake is through fortification of more frequently eaten foodstuffs with prebiotic ingredients. Prebiotics are thus a sub-category of functional food ingredients. They can be added to many foods including yogurts, cereals, breads, biscuits, milk desserts, ice creams, spreads, drinks as well as animal feeds and supplements. Galacto-oligosaccharides are another class of prebiotics that are manufactured and marketed in Europe as well as in Japan. These are successful prebiotics synthesised from lactose^(34)^. *In vivo* trials are the definitive assessment of a prebiotic effect, whether the target is the human situation or animals (livestock, pets). The hunt for new candidate prebiotics frequently explores oligosaccharides from different sources, including pectin and cellulose; starch and their breakdown product, maltose; xylan from wheat bran; mannose from fruits and vegetables; and the synthetically formed polydextrose, palatinose and lactulose^(35)^.

### Synbiotics

A synbiotic is a combination of the concepts of probiotics and prebiotics and consists of a live microbial food additive together with a prebiotic oligosaccharide (Table 1). Advantages are that a probiotic with known benefits can be used, and the prebiotic aids establishment of the organism in the complex colonic environment. This would be a synergistic synbiotic. On the other hand, the combinations used may act independently of each other – a complementary synbiotic. There is thus flexibility in the choice of live micro-organisms and substrate with the best combination for a specific desired outcome being determined^(36)^.

## Aspects of gut microbiota modulation related to respiratory infection and COVID-19

Immune changes brought about by the gut microbiota can influence respiratory conditions^(37)^. For example, evidence from studies using germ-free mice that are highly susceptible to numerous viral infections, including influenza, indicates that resident gut microbiota shapes anti-viral defences and modulates outcome of certain viral infections^(38)^. Indeed, differences in the gut microbial community have been demonstrated in other viral infections such as influenza and pneumonia^(39)^. Subsequent investigations of strategies to alter microbial changes have been seen to positively impact upon disease outcomes; some such studies are outlined below.

Numerous studies have focused on modulation of the gut microbiota and its impact on upper respiratory tract infections (URTI) resulting in three meta-analyses reporting that probiotics can reduce severity and duration of illness^(40–42)^, with similar findings for synbiotics^(43)^. Mechanistically, modulation of the gut microbiota has been demonstrated to increase positive bacteria whilst enhancing the activities of cytotoxic T-cells and T-suppressor cells^(44–46)^ or through supporting natural killer cell activity^(47)^. To illustrate this, De Vrese *et al.* ^(45)^ explored the use of probiotics in healthy adults aged 18–67 years. Four hundred and seventy-two volunteers were tested over two winter periods whilst consuming a probiotic mixture with *Lactobacillus gasseri* PA 16/8, *Bifidobacterium longum* SP 07/3, *Bifidobacterium bifidum* MF 20/5 plus vitamins and minerals, or a placebo of just the vitamins and minerals. Whilst the study resulted in volunteers on the probiotic being equally likely to pick up a respiratory infection as the placebo, there were 13·6 % fewer virally induced URTI in the probiotic treatment group^(48)^. Furthermore, those on probiotics who did develop URTI had a shortened duration of symptoms by 21·5% on average (improved recovery by on average 2 d), less severe symptoms and volunteers were less likely to develop fever, combined with elevated levels of CD4^+^ and CD8^+^. Such a decrease in symptoms and up-regulation of immune responses could lessen the impact of viral burden. For prebiotics, the use of fructans and glucans in infant formulae resulted in fewer (*P* < 0·01) episodes of physician-diagnosed overall and URTI (*n* 66) compared with controls without prebiotics (*n* 68)^(49)^. In another trial, Shahramian *et al.* ^(50)^ used galactans and polydextrose to reduce respiratory tract infections in formula fed infants (*n* 60) studied over 1 year (*P* = 0·01, compared with controls without prebiotics, *n* 60). Effects were comparable with those of breast-fed infants (*n* 60).

SARS-CoV-2 most commonly manifests as a URTI but can, in more severe cases, proliferate deeper into the lungs to become a lower respiratory tract infection (LRTI). Whilst evidence is still being collated, recent meta-analyses of randomised control trials have indicated that probiotics can reduce the incidence and severity of ventilator-associated pneumonia^(51,52)^. In a study by Mahmoodpoor *et al.* ^(53)^, for example, probiotic supplementation shortened the duration of ventilator use in critically ill patients. Furthermore, Shimizu *et al.* ^(54)^ issued synbiotics within 3 d of hospital admission where mechanical ventilation was used in sepsis patients. The intervention resulted in less ventilator-associated pneumonia (with 48·6–14·3 % of cases with no synbiotics to synbiotics, respectively), whilst increasing numbers of faecal *Bifidobacterium* spp. and *Lactobacillus* spp. Similar findings have been observed in other probiotic ventilator-associated pneumonia studies^(55)^ indicating that modulation of the gut microbiota may also have a part to play in LRTI.

Influence of the gut microbiota may be at least partially responsible for the strongest COVID-19 risk factors. Advancing age is a risk factor for COVID-19, with Worldwide data reporting deaths in 14·8 % of individuals over 80 years of age contracting COVID-19, contrasting with 8 % for those 70–79 years of age and 3·6 % in 60–69 years of age^(56)^. Populations of gut bacteria change with age^(57)^, for example, lower levels of bifidobacteria are associated with older populations^(58)^. These changes in composition of the gut microbiota may be a contributing factor to other age-related physiological changes such as reduced gut epithelial barrier function, poorer immune function and an increased inflammatory state (loosely termed ‘inflammageing’)^(59)^. This may be instrumental to the increased risk of infection observed in the elderly.

Alteration of the gut microbiota can also reduce inflammatory status in the elderly. Studies in older populations by ourselves, for example, have shown that prebiotic galacto-oligosaccharides can lead to enhanced bifidobacterial levels in older populations concurrent with increased anti-inflammatory IL-10 and reduced pro-inflammatory cytokines, including IL-6, IL-1β and TNF-α^(60,61)^. As an increased inflammatory state seems central to advanced COVID-19 manifestation, prophylactically reducing general inflammation could help support overall immune function.

Excess weight appears to be another risk factor for COVID-19. According to the Intensive Care National Audit & Research Centre report on COVID-19 in Critical Care of UK (27 March 2020)^(62)^, out of 775 patients, 72·1 % were overweight or obese; furthermore, 60·9 % of ICU patients who died were obese. Having looked at these data, Muscogiuri *et al.* ^(63)^ suggested that those with cardiometabolic conditions were more likely to be at a higher risk of a poorer COVID-19 prognosis.

Obesity is also associated with low-grade chronic inflammation, characterised by elevated levels of pro-inflammatory cytokines^(64)^. These changes are linked to increased circulating levels of endotoxin, which is a component of Gram-negative bacterial cell walls that normally remains separate from the blood system due to the epithelial barrier. In addition to this impact on inflammatory state, a poorer gut barrier which may be a facet of ageing can also enable passage of bacteria and viruses from the gut lumen to the blood which could lead to increased secondary infections in COVID-19 patients^(65)^. In this context, it has been reported that probiotic use can help improve barrier integrity in the gut^(66,67)^. Moreover, Luo *et al.* ^(68)^ also considered that modulation of the gut microbiota may help to avoid secondary infections by reducing micro-organism transfer to the gut. This is of relevance due to findings on COVID-19 and secondary infections. Zhuo *et al.* ^(69)^ in Wuhan indicated that, in a cohort of volunteers with COVID-19, 50 % of those who died had secondary bacterial infections.

In individuals with the metabolic syndrome, treatment with prebiotic galacto-oligosaccharides enhanced beneficial members of the microbial community, including bifidobacteria, whilst reducing markers of metabolic illness and levels of inflammation as determined by faecal calprotectin levels^(60)^. Furthermore, murine studies suggest that these effects are mediated in part through improvements in epithelial wall integrity mediated by the gut bacteria^(70,71)^. Thus, changes observed within gut microbiota modulation studies act to reduce inflammatory status and may reduce carriage across the gut epithelium. Such changes could act against hyper-inflammation and secondary infections.

## Is there a role for the gut microbiome and probiotics/prebiotics in the treatment of COVID-19?

In the current climate, a reduction in illness severity and duration could be an asset not only to National Health Services but also to those suffering with COVID-19. As mentioned, there is a body of evidence supporting a prophylactic role of probiotics, prebiotics and synbiotics in reducing symptoms with regard to URTI. Studies modelling the impact of probiotic consumption on respiratory infections in the pre-COVID-19 era highlight economic savings that could be brought about by probiotic consumption in the general population^(72,73)^. Given the potentially life-threatening nature of COVID-19, such studies are all the more pertinent now. It is worth noting that probiotics are accepted as safe in most situations and have been utilised without adverse effect in many trials with vulnerable individuals^(23,25)^.

**Fig. 1.**
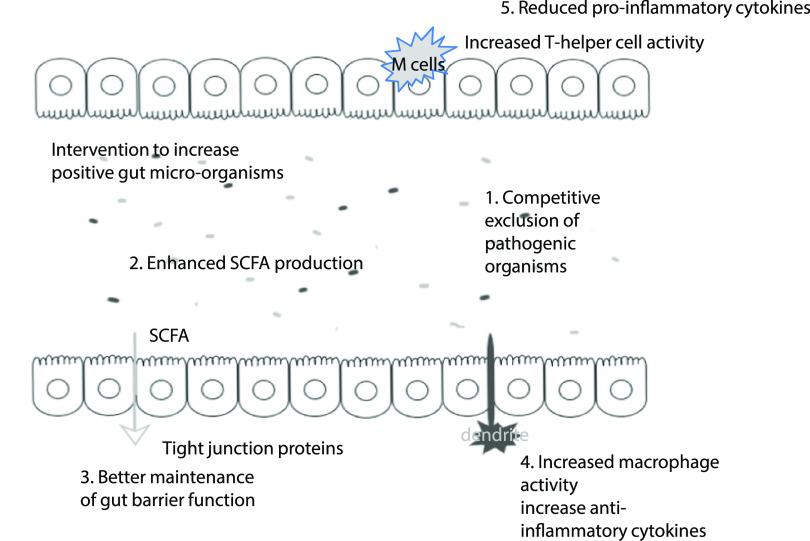
Possible pre- or probiotic mechanisms in the fight against COVID-19. Through increasing levels of positive micro-organisms in the gut, this can impact on a range of pathways that could be of benefit in the fight against COVID-19: (1) Intervention with pre- and probiotics positively alters the bacterial community in the gut, often to the detriment of potentially pathogenic micro-organisms (through the production of metabolites and/or competitive exclusion. This can reduce the risk of gut-related illness. (2) The gut microbiota produce SCFA that have systemic effects in the body, including provision of a cellular energy source. (3) Increased SCFA levels are associated with improved tight intestinal junctions, possibly restricting the passage of endotoxin from the gut lumen; these effects may be in part mediated by cytokines. Increased levels of bifidobacteria have also been associated with improvement of tight junctions. It is worth noting that both ageing and obesity (which are COVID-19 risk factors) are associated with a poorer gut barrier function. (4) Free fatty acid receptors can be found on dendritic cells, as such SCFA stimulation of dendrites can impact on regulatory T-cells resulting in enhanced macrophage activity and increased anti-inflammatory cytokines. (5) Macrophages are associated with pro-inflammatory status; however, SCFA have been observed to down-regulate pro-inflammatory cytokine release. By supporting the immune function and reducing inflammatory status, the gut microbiota could be a tool to aid the body in defending from COVID-19.

Mechanisms that might explain these positive gut modulating effects are through direct interaction with the intestinal immune and epithelial cells or indirect modulation by the intestinal microbiome. Beneficial effects include enhancement of the intestinal epithelial barrier, competition with pathogens for nutrients and adhesion to the intestinal epithelium, production of antimicrobial substances and modulation of the host immune system (both innate and adaptive)^(74)^, see Fig. 1.

Central to the maintenance of epithelial integrity and modulation of the immune system are SCFA. These are end products of microbial fermentation, particularly associated with breakdown of carbohydrates in the colon. SCFA, including acetate, propionate and butyrate, exert effects throughout the body^(75)^. SCFA have been observed to bind to free fatty acid receptors, which are highly expressed by immune function cells. Subsequently, SCFA have been observed to promote the development of naive CD4^+^ T-cells into regulatory T-cells^(76)^ and enhance cytotoxic T-cell, T-suppressor cell, CD8^+^ T-cell and natural killer cells activities^(44,47,77)^. Moreover, SCFA are involved in enhancing the immune response to pathogens via IL-18, defensins and toll-like receptors^(78,79)^. As such, enhancing the immune system to fight against infections could be a good weapon against COVID-19 and associated secondary infections.

Fibres have been shown to possess immunomodulatory properties^(80)^; in the case of infant gut microbiota, the prebiotic inulin has been observed, *in vitro*, to attenuate pro-inflammatory responses^(81)^, whilst Vogt *et al.* ^(82)^ found that inulin supplementation along with hepatitis B vaccination led to higher *in vitro* antibody titres compared with control intervention. Research by Trompette *et al.* ^(83)^ showed that high fibre levels in the diet of mice influenced the gut microbiota and SCFA levels. It was observed that allergic airway disease symptoms were greater in low fibre fed animals, whilst inclusion of SCFA in the diet negated this difference. Airway allergy disease is associated with inflammation. In the present study, via the gut microbiota, SCFA production led to reduced inflammation via free fatty acid receptors, therefore illustrating how the gut–lung axis could operate.

Research into respiratory syncytial virus of infants, a key cause of LRTI, has shown an altered microbial community to be associated with severe disease symptoms^(84)^. A recent review by Enaud *et al.* ^(85)^ documented current knowledge about the gut–lung axis. He *et al.* ^(86)^ discussed how ACE2 expression is down-regulated in SARS patients during infection. This is of interest in terms of the gut microbiota as ACE2 regulates expression of amino acid transporters that control intestinal uptake of tryptophan. Tryptophan regulates antimicrobial peptides, which could result in changes to the gut microbiota.

There is evidence supporting a common mucosal immunity, whereby the immune status of the gut is evidenced to affect other sites of the body^(87)^. As such, differences in the gut microbiota observed during COVID-19 could also be involved in gut–lung crosstalk^(88)^. Enaud *et al.* ^(85)^ detailed how probiotics may impact respiratory immunity. Probiotics have been demonstrated to improve levels of type I interferons, increase the number and activity of antigen presenting cells, NK cells and T-cells, plus systemic and mucosal specific antibodies in the lungs. Probiotics may also influence the balance between pro-inflammatory and immunoregulatory cytokines that allow viral clearance while minimising immune response-mediated damage to the lungs. The concept of gut–lung axis has also been illustrated during lung viral infections when symptoms are worse in antibiotic microbial-disrupted mice compared with colonised counterparts; these differences have been mitigated by probiotic treatment in antibiotic treated animals^(89)^. The observed effects were a result of the gut microbiota up-regulating expression of toll-like receptor 7 influenza-infected macrophages, supporting the immune response.

Further evidence of the gut–lung axis in action is illustrated in the research of Haak *et al.* ^(90)^. Faecal samples were taken from 360 allogeneic hematopoietic stem cell transplant patients (these patients often develop respiratory infections). Within the patients following transplant, 41 % developed viral respiratory infections and 31·5 % developed LRTI. When correlating with the microbiota, it was observed that those with higher levels of butyrate producing bacteria were five times less likely to develop LRTI. As a word of caution, probiotic intake in those in intensive care does require more research to establish safety protocols across the range of available probiotics and the different pathological conditions that require intensive care.

Clinicaltrials.gov currently reveals nine trials exploring the impact of probiotics on COVID-19; these include a prophylactic focus on *Lactobacillus rhamnosus* GG in household contacts of COVID-19 sufferers (Wischmeyer and Sung, North Carolina)^(91)^ and two studies (Spain and Brussels) on health care personal/professionals (Rodriguez Blanque, Kenz)^(91)^. The other studies focus on those already with symptomatic COVID-19 (non-ICU) to assess changes in symptoms (Navarro, Desrosiers, Pugliese, Graz, Gea Gonzalez, Sapienza and Saralaya)^(91,92)^.

In an exciting development, one newly completed study published by d’Ettorre *et al.* ^(93)^ examined seventy patients positive for COVID-19 requiring non-invasive oxygen therapy who were provided with hydroxychloroquine therapy along with antibiotics and tocilizumab; in twenty-eight of these, an oral probiotic mixture was also administered. Along with improved gut symptoms, the probiotic group had an eight-fold reduction in risk of developing respiratory failure. This shows much promise and data evidence for the use of probiotics to combat respiratory difficulties. As clinical evidence gathers, the role for a simple and safe prebiotic or probiotic intervention against COVID-19 infection could become more important.

Also of note, Cao *et al.* suggested that polysaccharides within a lung cleansing concoction were likely to be an effective approach for managing mild COVID-19 symptoms, and this was considered to be due to gut microbiota modulation and immune function supporting roles of these ingredients^(94)^. This suggests that prebiotics may be used to support the gut microbial composition and aid against secondary bacterial infection in these patients.

It is also worth noting that differences in intestinal microbiome may compromise the effectiveness of vaccine antigens as a consequence of chronic inflammation of the intestinal tract^(95)^. Indeed, meta-analysis of pre- and probiotic studies has concluded that intervention alongside influenza vaccination can lead to elevated immunogenicity through enhancing sero-conversion of inoculated persons^(96–98)^. Dietary intervention, therefore, may be an important prerequisite before vaccination against COVID-19 particularly in those at risk of an altered gut microbiota such as individuals with metabolic disorders and the elderly.

In China, recommendations have been made with regard to modifying the gut microbiota to improve outcomes in patients with severe COVID-19 symptoms^(99)^. These recommendations were based on observed differences in the faecal microbiota in those with COVID-19 compared with healthy controls which suggested that the virus can replicate and exist in the digestive tract^(100)^. Additionally, Pan *et al.* ^(101)^ reported the presence of gastrointestinal symptoms in half of COVID-19 patients within a group of hospitalised Chinese patients with disease severity correlating with the severity of gut symptoms. This a clear demonstration that, in China at least, the gut microbiota is considered to be an important influencer on COVID-19 outcome.

In conclusion, there is currently clinical evidence gathering to indicate that modulation of the gut microbiota can positively influence COVID-19 disease progression. This is further supported by reported positive effects of probiotics against other coronavirus strains^(102)^. Studies are underway across the globe to investigate whether altering the gut microbiota through diet might be a feasible addition to our COVID-19 treatment armoury and recently, Baud *et al.* ^(103)^ have suggested specific evidence-based probiotic products that may have relevance to reducing the coronavirus pandemic burden.

The race for a vaccine and pharmaceutical treatments for the current COVID-19 pandemic continues. However, both are likely to be some way from routine use and, in the meantime, attention should be given to emerging, but convincing, evidence that gut health may be related to COVID-19^(12,104–106)^. The approaches suggested here to improve gut microbial health are safe and straightforward to implement and have a scientific basis. In the current climate, a reduction in illness severity and duration could be an asset not only to health systems worldwide but also to those suffering with COVID-19.
